# Reversed cortical over-activity during movement imagination following neurofeedback treatment for central neuropathic pain

**DOI:** 10.1016/j.clinph.2016.06.012

**Published:** 2016-09

**Authors:** Muhammad Abul Hasan, Matthew Fraser, Bernard A. Conway, David B. Allan, Aleksandra Vučković

**Affiliations:** a Rehabilitation Engineering and Assistive Technologies, Biomedical Engineering Research Division, University of Glasgow, Glasgow, UK; b Department of Biomedical Engineering, NED University of Engineering and Technology, Karachi, Pakistan^1^; c Queen Elizabeth National Spinal Injuries Unit, Southern General Hospital, Glasgow, UK; d Department of Biomedical Engineering, University of Strathclyde, UK

**Keywords:** Central neuropathic pain, Neurofeedback, EEG, Motor imagery, Theta band

## Abstract

**Objective:**

One of the brain signatures of the central neuropathic pain (CNP) is the theta band over-activity of wider cortical structures, during imagination of movement. The objective of the study was to investigate whether this over-activity is reversible following the neurofeedback treatment of CNP.

**Methods:**

Five paraplegic patients with pain in their legs underwent from twenty to forty neurofeedback sessions that significantly reduced their pain. In order to assess their dynamic cortical activity they were asked to imagine movements of all limbs a week before the first and a week after the last neurofeedback session. Using time–frequency analysis we compared EEG activity during imagination of movement before and after the therapy and further compared it with EEG signals of ten paraplegic patients with no pain and a control group of ten able-bodied people.

**Results:**

Neurofeedback treatment resulted in reduced CNP and a wide spread reduction of cortical activity during imagination of movement. The reduction was significant in the alpha and beta band but was largest in the theta band. As a result cortical activity became similar to the activity of other two groups with no pain.

**Conclusions:**

Reduction of CNP is accompanied by reduced cortical over-activity during movement imagination.

**Significance:**

Understanding causes and consequences mechanism through which CNP affects cortical activity.

## Highlights

• Long-term neurofeedback treatment reduced central neuropathic pain and cortical overactivity in painful paraplegia.
• Reduction of event related desynchronization induced by movement imagery was largest in the theta band.
• This effect was strongest during imagined movements of painful and paralysed legs.

## 1 Introduction

Central neuropathic pain (CNP) is a frequent secondary consequence of spinal cord injury (SCI), affecting about 40% of patients (Siddall et al., 2003). Although CNP is caused by an injury to the somatosensory system (Haanpää et al., 2011) it can show first symptoms years after SCI. Neuroimaging studies have demonstrated changes in the resting state brain activity in the presence of CNP, which is reflected in increased thalamo-cortical coherence in the theta band (Stern et al., 2006, Sarnthein and Jeanmonod, 2008), as well as increased resting state EEG power and a dominant alpha frequency shift towards lower frequencies (Stern et al., 2006, Sarnthein et al., 2006, Boord et al., 2008, Jensen et al., 2013a, Vuckovic et al., 2014).

Both functional magnetic resonance imaging (fMRI) and electroencephalographic (EEG) studies (Gustin et al., 2010, Vuckovic et al., 2014) reviled that the increased activation and reorganisation of the sensory-motor cortex is a distinctive signature of CNP. During imagined movements of a paralysed limb (perceived as being painful) patients with SCI and CNP show the activation of brain areas related to both motor imagination and pain processing (Gustin et al., 2010). In an EEG study by our group that included SCI patients with and without CNP and healthy controls (Vuckovic et al., 2014), we demonstrated that during imagination of movement, patient with CNP had stronger event related desynchronization (ERD) (Pfurtscheller and Lopes da Silva, 1999) the healthy controls, while patients with no pain had weakest responses of all three groups.

A recent Cochrane study (Boldt et al., 2014) lists a number of non-pharmacological non-invasive treatments of CNP for SCI patients. Most of these studies comprise of up to 10 treatment sessions, which might not be long enough to induce longer lasting cortical changes; in addition, the assessment of brain activity has not been included in the outcome measures in none of the studies. Lefaucheur et al. (2006) showed that a single dose of rTMS which reduced symptoms of CNP also restores intracortical inhibition, but does not affect the excitability of the motor cortex, as measured by the amplitude of the motor evoked potential. It is however not known, what is the effect of a prolonged treatments of CNP on closely related, altered activity of the sensory-motor cortex.

Neurofeedback is a non-invasive technique which relies on measuring brain activity in real time. It has proved useful for treatments of different types of chronic pain, including CNP (Jensen et al., 2013a, Hassan et al., 2015). Neurofeedback trains a person to change his/her brain activity at will that can lead to the reduced sensation of pain. Thus it enables a direct voluntary modulation of the activity of the cortical regions that have been affected by pain. The ability to self-regulate brain activity is what makes this technique unique compared to other neuromodulation approaches such as Transcranial Magnetic Stimulation (rTMS) or transcranial Direct Current Stimulation (tDCS) (Boldt et al., 2014) in which a patient passively receives an external stimulus that modulates cortical activity.

In our recent study, 5 patients with SCI and CNP received from 20 to 40 daily neurofeedback treatments (Hassan et al., 2015). They achieved 25% and larger reduction of pain that was accompanied with changes in the resting state EEG power, in pain-related areas of the cortex. Although resting state network of sensory-motor cortex has a close relationship with the task related brain activity (Ma et al., 2011, Várkuti et al., 2013), the activity of the motor cortex, which is uniquely related to this type of chronic pain, is best assessed during a motor task (Gustin et al., 2010).

In this study we test a hypothesis that the reduction of pain intensity is accompanied with reduced activation of the sensory-motor cortex during imagined movements.

## 2 Methods

### 2.1 Participants

A total of 25 volunteers were recruited in 3 age-matched groups:

• Group1. Five paraplegic patients with diagnosed CNP below the level of injury (age 50 ± 4, 6 males, 1 females) here called PWP (patients with pain).
• Group 2. Ten paraplegic patients with no chronic pain (age 44.4 ± 8.1, 8 males, 2 females) here called PNP (patients with no pain).
• Group 3. Ten able-bodied volunteers with no chronic pain (age 39.1 ± 10.1, 7 males, females), here called AB (able bodied).

The neurological level of injury in patient groups was determined using the American Spinal Injury Association (ASIA) impairment classification (Marion et al., 2013). All patients were at least one year post-injury with a spinal lesion at or below T1. Inclusion criteria for patients with CNP were a pharmacological treatment history for at least 6 months and a reported pain level equal or larger than 5 on the Numerical Pain Rating Scale (NPRS 0 = no pain, 10 = worst pain imaginable). Patients in PWP group were asked not to change pharmacological pain treatment during the study.

**Fig. 1:**
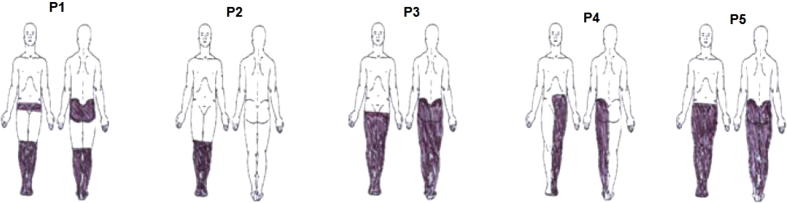
Location of perceived pain as indicated by patients who received neurofeedback training.

A general exclusion criteria were brain injury or other neurological conditions that might affect EEG and the presence of any chronic or acute pain at the time of the experiment. Group PWP is a subgroup of patients reported in (Vuckovic et al., 2014) that underwent neurofeedback training, described in (Hassan et al., 2015). Information about PWP patients and the outcome of the treatment can be found in Table 1. Information about PNP group is shown in Table 2. Fig. 1 shows the location of perceived pain (note that in the case of neuropathic pain the source of pain is actually not in the limbs but in the brain). Pain was typically described as stabbing, burning or squeezing.

An informed consent was obtained from all participants, and ethical approval for patients was obtained from the National health service regional Ethical Committee and for able-bodied volunteers from the University Ethical Committee.

### 2.2 The experimental protocol

A detailed experimental protocol is provided in Vuckovic et al. (2014) and here we provide a brief description. All groups followed the same protocol and PWP group performed the same experiment twice, first time about a week before the first neurofeedback session and second time about a week after the last neurofeedback session. Other two groups performed the experiment on one occasion only as they did not take any neurofeedback training.

**Fig. 2:**
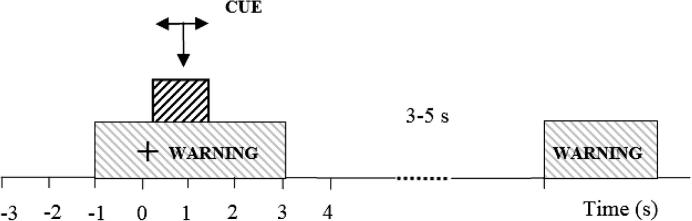
Experimental setup: at *t* = −1 s a readiness cue (a cross) appeared on a computer screen, followed by an execution cue (an arrow) at *t* = 0 s. The execution cue stayed on the screen till *t* = 1.25 s while the warning stayed until *t* = 3 s. A volunteer was asked to perform repetitive imagination of movement from *t* = 0 s till the readiness cue disappeared at *t* = 3 s. Different arrows indicate motor imagination of different limbs.

Standard cue-based motor imagination experimental protocol was used (Neuper et al., 2006). Precise cueing was necessary because of the lack of muscle activity while people imagined movement. The purpose of motor imagination was to induce activity of the cortico-spinal tract, thus serving a similar purpose as single pulse TMS as in (Lefaucheur et al., 2006). We were however primarily interested in modulation of the activity of the motor cortex, therefore we measured EEG responses. Participants sit approximately 1.5 m in front of a computer monitor. They were instructed to look at the center of the monitor and to respond to a sequence of visual cues. The cues included a readiness cue (presented as a cross) at *t* = −1 s and remaining on for 4 s (Fig 2). At *t* = 0 s an initiation cue (presented as an arrow), was displayed for 1.25 s, pointing to the right, to the left or down and corresponding to the imagination of the right and left hand waving, and tapping with both feet. Participants were asked to continue to perform imaginary movements for 3 s, until the cross disappeared from the screen. In total, 60 trials of each movement type were presented to subjects, 180 trials in total. Cues were presented in smaller sub-sessions, in randomized sequences comprising 10 trials of each movement with rest periods between.

### 2.3 EEG recoding and analysis

A 61-channel EEG was recorded (Synamp 2; NeuroScan, Charlotte, NC) with electrodes placed according to 10–10 location standard (ACNS, 2006). Electrode location AFz was used as a ground, combined with a linked ear reference. All channels were sampled at 1000 Hz and electrode impedance was kept below 5 kΩ. Electrooculogram was recorded around the right eye from 3 channels. Electromyograms (EMGs) were recorded from the right shank and from the left and right wrist extensor muscles using bipolar inputs to the Synamp device. The purpose of EMG recording was to check for the absence of voluntary movements when subjects imagined to move.

For off-line analysis a high pass filter (IIR, 12db cut-off frequency) was set to 1 Hz. To remove line noise at 50 Hz, a notch filter was applied between 48 and 52 Hz. Filtering was applied twice, forwards and then backwards to avoid a phase shift and was followed by down-sampling to 250 Hz. Independent component analysis (Bell and Sejnowski, 1995) based on the Infomax algorithm implemented in EEGLab (Delorme and Makeig, 2004) was performed for advanced noise removing purposes. In this was we avoided excessive EEG removal from a limited number of trials, removing no more than 2 (out of 60) trials per data set. EEG data were referenced to the average reference before performing further analysis.

EEG analysis was performed on a group level, by designing two studies with two variables: groups and experimental conditions. The first study design had two groups: patients with pain before a neurofeedback treatment (PWP_before) and patients with pain after neurofeedback treatment (PWP_after). There were three identical conditions in each group: imagined movement of the right hand, left hand and feet.

The second study design comprised of four groups: AB, PNP, PWP_before and PWP_after. Three ‘conditions’ corresponded to three type of imagined movement, the same as in study design 1. All analysis was performed in EEGlab.

### 2.4 Event-related synchronization/desynchronization phenomena as a measure of the activity of the sensory-motor cortex

Data analysis was based on a phenomena called Event Related Synchronization/Desynchronization (ERS/ERD) (Pfurtscheller and Aranibar, 1977, Pfurtscheller and Lopes da Silva, 1999), visible in time–frequency domain. During imagination or real execution of limb movements, neuronal firing is desynchronized, and as a consequence the amplitude and energy of the measured EEG signal decrease, as compared to the energy level in the reference period before the movement. This phenomenon is called event-related desynchronization (ERD), while opposite phenomenon, increased synchrony resulting in increased energy level, is known as event-related synchronization (ERS). A typical ERD/ERS sequence involves ERD in the alpha and some portion of the beta band starting during preparation of movement, often lasting throughout the movement. Following termination of movement, this is followed by ERS in the beta band. The method requires that power of EEG signal is collapsed over a pre-defined frequency band (Pfurtscheller and Aranibar, 1977, Pfurtscheller and Lopes da Silva, 1999, Neuper et al., 2006).

For a chosen frequency band, in its simplified version, a formula for ERS/ERD is

(1) $$\text{ERS}/\text{ERD}\;(\%)=\frac{E-R}{R}$$

where *E* is ‘an event’, e.g. imagination and *R* is ‘a reference period’ preceding imagination of movement. An extension of the ERS/ERD, called Event Related Spectral Perturbation (ERSP), based on Morlet wavelets (Makeig, 1993), was used to allow simultaneous analysis of ERS/ERD phenomena on multiple frequencies which present ERS/ERD in a form of time–frequency maps. Although we present ERSP rather than ERS/ERD in isolated frequency bands, to make a distinction between ERS and ERD phenomena, the method will be called ERS/ERD further in the text. Time–frequency decomposition was performed in a frequency range 3–55 Hz. A minimum 3 wavelet cycles per data window was used at lowest frequencies that allowed low frequencies starting from 3 Hz to be analysed in one second window. The number of cycles increases to 14 on highest, logarithmic spaced frequencies providing a better frequency resolution at higher frequencies (Delorme and Makeig, 2004). For calculating the ERS/ERD of each single volunteer, a reference period was adopted from a period before the readiness cue.

### 2.5 Statistical analysis

In order to find the regions of significant ERS/ERD in a time–frequency map for a single electrode site, a significance level was set to *p* = 0.05 and a nonparametric bootstrapping procedure (*N* = 2000 trials) (Blair and Karniski, 1993) was performed, comparing ERD/ERS maps between groups. Because a time–frequency map results in multiple time–frequency windows in time–frequency space and bootstrapping test is performed for each window independently, a False Discovery Rate (FDR) correction was applied, to correct the significance level for multiple comparisons (Benjamini and Yekutieli, 2001).

Statistical analysis, applying the aforementioned method was performed on (a) ERS/ERD maps of individual patients before and after neurofeedback therapy, (b) ERS/ERS on a group level for patients with pain before and after neurofeedback therapy, (c) ERS/ERS between different groups of volunteers.

Scalp ERS/ERD maps were created by averaging over theta, alpha and beta 16–24 Hz frequency bands (*F*) and short time windows (*T* = 400 ms). For each electrode this provided an average ERS/ERD value for each T*F window. Based on 61 averaged values, one for each electrode location, a topographical two dimensional scalp distribution of ERS/ERD was interpolated using spline interpolation method (Perrin et al., 1989). A comparison between the scalp maps of different groups or conditions was performed based on a permutation statistics (*p* = 0.05) as previously described and FDR was applied to account for comparison from multiple electrode sites. All calculations were performed in EEGlab toolbox for Matlab (Mathworks Inc. USA).

### 2.6 Neurofeedback training

Neurofeedback training and results are described in detail in Hassan et al. (2015). Although we do not present results of neurofeedback training session, here we explain the training technique and rationale for a chosen protocol. We used operant conditioning neurofeedback technique, based on non-verbalised rules, in which a person learns how to modulate his/her behavior based on video or audio feedback information about the consequences of that behavior. This technique has been used for treatment of various conditions including attention deficit hyperactivity disorder, epilepsy or depression (Laibow, 1999, Demos, 2005). It should not be confused with neurofeedback based on a verbalised strategy (strategy that can be explained with words) such as motor imagery (imagination of movements), that has been extensively used in the area of Brain Computer Interface (Gomez-Pilar et al., 2016).

Although we used cue-based motor imagery for the assessment purposes, and we defined cortical areas and frequency bands which are affected by pain, we could not use motor imagery as a neurofeedback strategy because, as described later in the text, we modulated some of brain features in a direction which was opposite form the direction of modulation during motor imagery. Furthermore, Gustin et al. (2008) showed that prolonged motor imagery can potentially increase pain.

During neurofeedback EEG was recorded with 256 samples/s, ground electrode was placed on the mastoid of the training side and the reference electrode on the mastoid of the opposite side. Impedance was kept under 5 kΩ and training was provided from one electrode at the time while EEG was recorded with up to 16 electrodes (Hassan et al., 2015). Recording was performed with usbamp (Guger Technologies, Austria) which has a proprietary software modules g.RTanalyzer for Simulink/Matlab that enable real-time EEG signal analysis.

At the beginning of each training session patients sit still and relaxed with their eyes open for 2 min while their EEG was recorded. This recording served as a baseline for a subsequent training. Patients were trained to increase the relative alpha band power (in this case 9–12 Hz) or low beta (12–15 Hz) power for 10% or more above the baseline value and to decrease the theta band (4–8 Hz) and the higher beta band (20–30 Hz) power for 10% or more under the baseline value. As explained in Hassan et al. (2015) neurofeedback training with increasing low beta (12–15 Hz) while decreasing theta and high beta, had no effect on the intensity of pain and was mostly practiced with the first patient, therefore it will not be discussed further. The main training protocol therefore consisted of increasing the alpha band power while simultaneously decreasing theta and higher beta band power.

Power was calculated by band-pass filtering EEG signal, smoothed/averaged over 0.5 s sliding window that was updated every 8 samples (about 30 ms) (Hassan et al., 2015). For normalization purposes, relative power was calculated by dividing the power of a chosen frequency band (theta, alpha or beta) with the total power in 2–30 Hz band. Patients were trained to control a relative power.

During neurofeedback training session three bars were presented on a screen, the middle, and the largest one, presenting the alpha power, a bar on the left always presenting the theta band power and the one on the right always presenting the high beta power. A visual presentation with bars is a standard graphical user interface used in commercially available neurofeedback devices (e.g. Mind Media, NeXus, USA). Participants were explained that they should primarily concentrate on the middle, largest bar. The bars changed its size and color (red or green) depending on the power of the representative frequency band. The only instruction given to patients was keep the bars green. Over a period of several daily sessions they came up with their preferred mental strategy. The middle bar turned green when the amplitude increased while the side bars turned green when their amplitude decreased. One daily training session lasted about 30 min was divided into shorter 5 min long sub-sessions.

A detailed rationale for choosing a specific frequency band and training protocols can be found in Hassan et al. (2015), and is related to both changes in EEG during resting state and during motor imagery. Here we provide a brief explanation: increased resting state theta band power is a confirmed signature of CNP in resting state EEG (Sarnthein et al., 2006, Boord et al., 2008, Jensen et al., 2013b, Vuckovic et al., 2014) and in (Vuckovic et al., 2014) we found that only patient with CNP show theta ERD during imagination of movement. Dominant alpha band frequency is shifted towards lower frequencies in patients with CNP (Sarnthein et al., 2006, Boord et al., 2008, Vuckovic et al., 2014) therefore we trained patients to increase the power of a slightly higher alpha band (9–12 Hz) with the idea of shifting dominant frequency toward higher values. The idea to increase the alpha power was based on Gustin et al. (2008) study showing that primary motor cortex is overactive in spinal cord injured patients with CNP. As alpha power typically decreases in the active state of motor imagery we trained patients to modulate the alpha activity in the opposite direction. Finally higher beta (20–30 Hz) power a showed positive correlation with chronic pain in previous studies (Sarnthein et al., 2006) therefore we trained patients to decrease power in this band.

Training was provided from the centro-parietal region (C4, C3, P4). Details on the exact training protocol diary for each patient, rationale of electrode selection and patient response are provided in Hassan et al. (2015). Electrode C4 and C3 are located over the primary motor cortex while P4 is located parietally, closer to the sensory cortex. Patients were most responsive to training from C4 (located above the primary motor cortex of the left hand) which was used for providing neurofeedback in most of training sessions for all patients. Training from location P4 resulted in reduced pain but less than training from C4. Training from C3 resulted in the comparable reduction of pain as C4 but produced in several patients increased spasm during training. We have not tested P3 (contralateral from P4) to minimize patient discomfort in case of possible spasm noticed during training from that hemisphere. Although patients had pain in their legs (motor cortex located around Cz) we found that somatotopic matching of modulation site with motor cortex of a limb with perceived pain was not necessary. We showed that changes of brain activity due to pain were wide-spread over the motor cortex of both painful and non-painful limbs (Vuckovic et al., 2014). This was also in-line with results of studies using rTMS and tDCS, which did not match stimulation site of the primary motor cortex with location of ‘perceived’ pain (Lefaucheur et al., 2008).

## 3 Results

We show a difference in cortical responses in PWP before and after therapy (PWP_after – PWP_before) in the form of a time–frequency response for individual electrodes and in a form of ERS/ERD scalp maps. Following that, we compare ERS/ERD time–frequency response over the primary-motor cortex between AB, PNP and PWP group before and after neurofeedback therapy.

### 3.1 The effect of neurofeedback training on ERS/ERD during motor imagination task in patients with pain

**Fig. 3:**
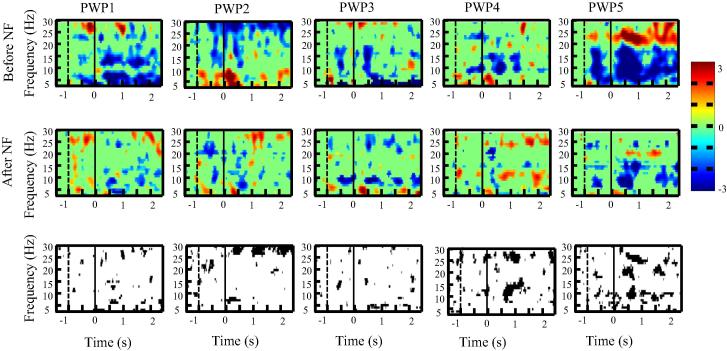
ERD/ERS time frequency map at C4 for each patient with pain (PWP1–PWP5) before and after neurofeedback training during motor imagination of the left hand (the upper and middle row consecutively). Subfigures at the bottom row show the areas of statistically significant (*p* < 0.05) difference in ERD/ERS before and after neurofeedback. The moment when a warning cue was presented is shown with a dashed line (*t* = −1 s) and the moment when an execution cue was presented is shown with a solid vertical line (*t* = 0 s).

**Fig. 4:**
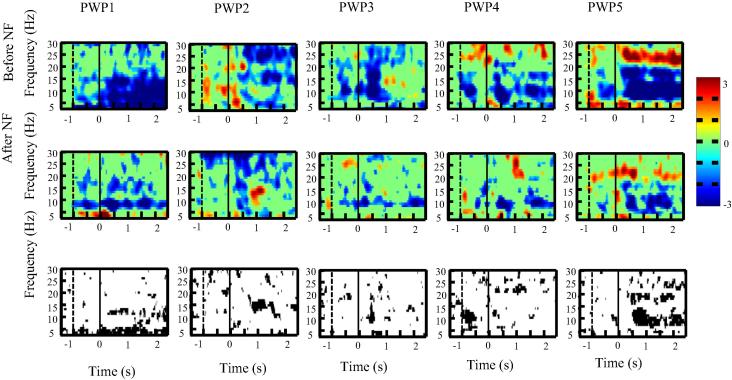
ERD/ERS time frequency map at Cz for each patient with pain (PWP1–PWP5) before and after neurofeedback training during motor imagination of feet (the upper and middle row consecutively). Subfigures at the bottom row show the areas of statistically significant (*p* < 0.05) difference in ERD/ERS before and after neurofeedback. The moment when a warning cue was presented is shown with a dashed line (*t* = −1 s) and the moment when an execution cue was presented is shown with a solid vertical line (*t* = 0 s).

**Fig. 5:**
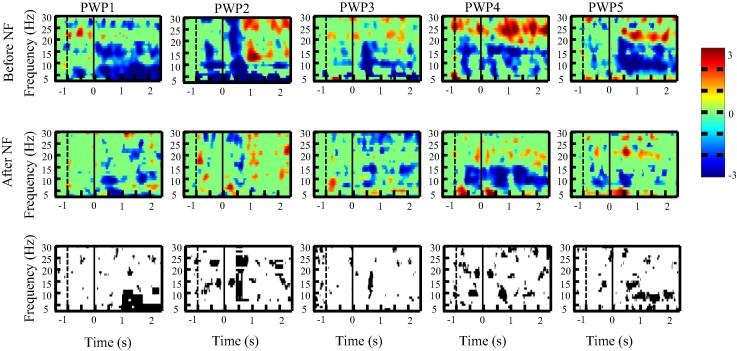
ERD/ERS time frequency map at C3 for each patient with pain (PWP1–PWP5) before and after neurofeedback training during motor imagination of the right hand (the upper and middle row consecutively). Subfigures at the bottom row show the areas of statistically significant (*p* < 0.05) difference in ERD/ERS before and after neurofeedback. The moment when a warning cue was presented is shown with a dashed line (*t* = −1 s) and the moment when an execution cue was presented is shown with a solid vertical line (*t* = 0 s).

We present first ERD/ES in the time–frequency domain over the primary motor cortex for each single patient (Fig. 3, Fig. 4, Fig. 5) to demonstrate consistency of results across patients.

Fig. 3, Fig. 4, Fig. 5 shows ERS/ERD maps for electrode locations Cz, C3 and C4 for all five patients before and after the neurofeedback treatment. All patients had paralysed legs and had perceived sensation of pain in their legs. In the upper two rows in Fig. 3, Fig. 4, Fig. 5, a darker color shows more ERD (e.g. effectively more activity) while a lighter color presents more ERS. Time is presented on *x* axis while *y* axis corresponds to frequency. A vertical dashed line at *t* = −1 s marks a moment when a warning sign was presented while a vertical sold line at *t* = 0 s marks a moment when an execution cue was presented on a computer screen. The bottom row in Fig. 3, Fig. 4, Fig. 5 shows a statistically significant difference in ERS/ERD between PWP_before and PWP_after.

Changes in ERS/ERD following prolonged neurofeedback could be caused by two factors: genuinely reduced activity (reduced ERD) during motor imagination (affecting value *E* in Eq. (1)) or by changes in the baseline that would affect value *R*. If neurofeedback training affects the baseline EEG power, then increased power would increase *R* leading to smaller ERD during motor imagination and decreased power would have the opposite effect. Patient 1 was most successfully in reducing theta band power, patients 2–4 were successful in increasing alpha and decreasing beta band power while patients 5 could regulate simultaneously all three frequency bands during neurofeedback (Hassan et al., 2015).

Fig. 3 shows ERS/ERD maps over the electrode location C4, located over the primary motor cortex of the left hand, while patients imagined to wave with their left hand. This was the electrode locations from which neurofeedback was provided in most of training sessions. Note that due to the numerical restriction of ERD/ERS analysis using wavelets, the last half second (*t* = 2.5–3 s) could not be displayed.

In all five patients, both alpha and beta ERD was reduced following neurofeedback training. Reduction of alpha ERD could be attributed to increased baseline due to upregulating alpha band power during neurofeedback training. The reduction of beta ERD is not related to the baseline power, as this was the only band that showed consistent baseline reduction of power across all patients (Hassan et al., 2015), This means that *R* value in Eq. (1) is smaller which should result in larger ERD. Therefore reduction in beta ERD is more likely to be the consequence of reduced deactivation during motor imagery, i.e. reduced parameter *E*.

Fig. 4 shows ERS/ERD over the electrode location Cz (located over the primary motor cortex of legs) while patients imagined tapping with both feet for 3 s. All patients had paralysed legs and reported sensation of pain in their legs. It can be noticed that before neurofeedback therapy, in all patients, ERD was spread over a wide range of frequency bands. After neurofeedback, ERD became confined to two narrow frequency bands in the alpha and beta bands, called ‘sensory motor rhythms’ (Pfurtscheller and Lopes da Silva, 1999, Niedermeyer, 2005). The ERS/ERD maps in PWP_after remind therefore on a typical response seen in able-bodied people with no pain (Pfurtscheller and Lopes da Silva, 1999). The reduction of theta band ERD is strongest at Cz compared to C3 and C4. Patients trained to downregulate theta band power so similar to beta band, reduced ERD is more likely to reflect reduced activation (reduced ERD) during motor imagination than a shift in baseline activity.

Fig. 5 shows ERS/ERD maps over the electrode location C3, placed over the primary motor cortex of the right hand, while patients imagined to wave repeatedly for 3 s with their right hand. Patients had no sensory-motor problems with their upper limbs and had no pain. Still, the intensity of ERD was reduced in all patients following neurofeedback treatment. In PWP4 the ERD frequency changed from the higher beta before neurofeedback to the lower beta and the alpha following neurofeedback treatment. In most patients, before treatment, ERD could be noticed in well-defined narrow frequency bands throughout the trial (from *t* = 0 till *t* = 3 s). Following the neurofeedback treatment, a statistically significant ERD can be noticed mostly within the first second (from *t* = 0 s to 1 s) and in a period *t* > 2 s close to the termination of movement.

The overall conclusion is that neurofeedback resulted in reduced ERD for both painful/paralysed and non-paralysed limbs. Changes were likely to reflect combined effect of changes in baseline EEG power (in alpha band) and reduced intensity of activation during motor imagery, i.e. both parameters *E* and *R* in Eq. (1).

**Fig. 6:**
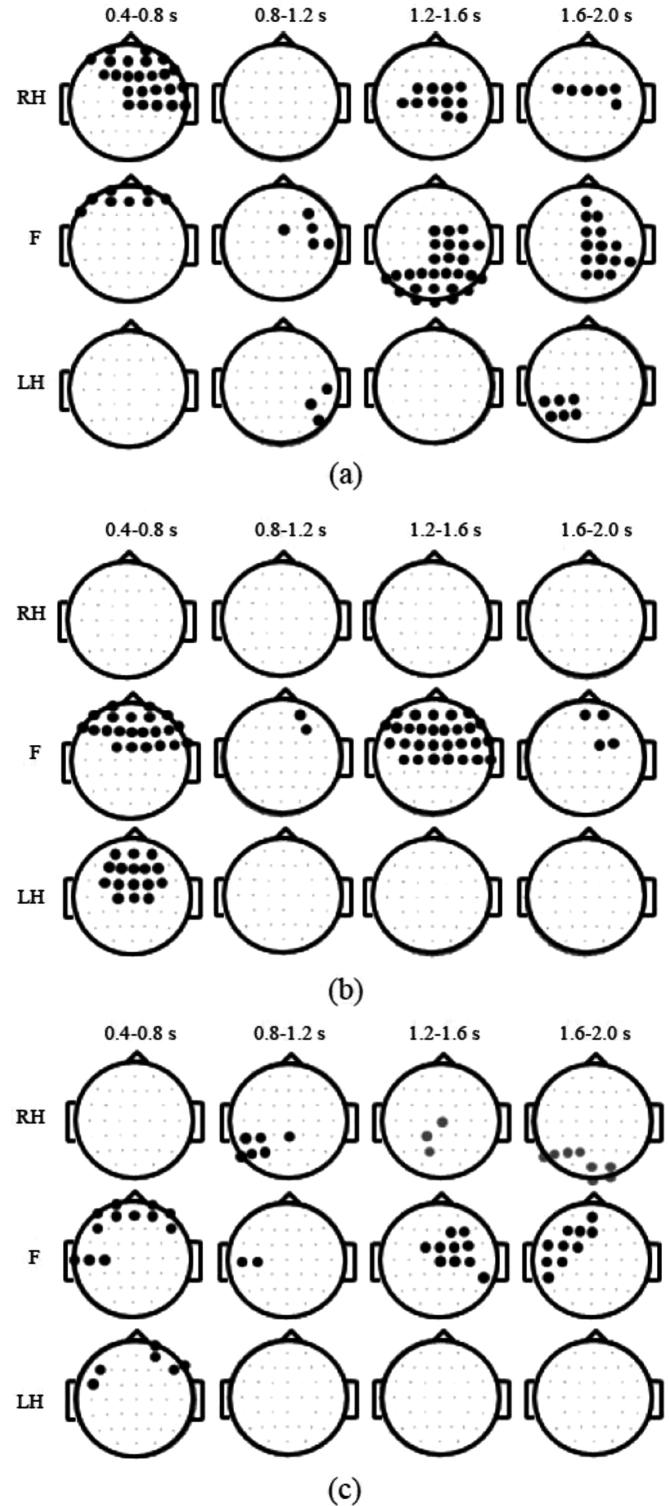
Comparison of spatio-temporal changes in ERD/ERS in PWP group (patients with pain) before the first and after the last day of neurofeedback training. Averaged group scalp maps showing statistically significant ERS/ERD between ‘Before NF’ and ‘After NF’ conditions in three frequency bands (theta, Fig. 5a; alpha, Fig. 5b; beta (16–24 Hz), Fig. 5c) averaged over four different time windows (0.4–0.8 s, column 1; 0.8–1.2 s, column 2; 1.2-1.6 s, column 3; 1.6–2.0 s, column 4) for three types of motor imagination tasks (RH; right hand, F; foot, LH; left hand). The black filled circles represent significantly reduced ERD, grey filled circles represent increased ERD, and smaller dots represent electrode locations with non-significant change in ERD/ERS, *p* < 0.05, corrected for multiple comparison.

We also observed scalp maps of ERD/ERS averaged over 400 ms periods from *t* = 400 ms to *t* = 2000 ms. Time windows earlier than 400 ms were not analysed because they are considered to be too early to present conscious actions. Fig. 6 presents a top view of a head, with the nose to the front. Electrodes marked in bold show locations with a statistically significant difference (*p* = 0.05) between ERS/ERD before and after neurofeedback, with applied corrections for multiple comparisons. A black dot means that the intensity of ERD was reduced following neurofeedback treatment while a grey dot means that the intensity of ERD was increased. We analysed three selected frequency bands, theta, alpha/mu (8–12 Hz) and beta (16–24 Hz), shown if Fig. 6a–c. The latter two frequency bands are typical frequencies of the sensory-motor rhythms (Niedermeyer, 2005) and are most reactive to imagined or executed movements.

Fig. 6a shows scalp maps for statistically significant differences in ERD in the theta band. Changes in theta activity over the large number of electrode locations can be noticed for motor imagination of feet and of the right hand. During movement preparation and initiation (*t* = 0.4–0.8 s) larger changes can be noticed in the frontal area, which is related to movement planning. In the later stage (*t* = 1.2–2.0 s) larger changes can be noticed over the centro-parietal cortex, an area responsible for spatial rotation, sensation and movement execution (Bear et al., 2007, Osuagwu and Vuckovic, 2014).

In the alpha band (Fig. 6b), largest reduction can be noticed in two time windows, *t* = 0.4–0.8 s during movement initiation and *t* = 1.2–1.6 s during sustained movement imagination. There were no changes in ERD for motor imagination of the right hand. Significant decrease in ERD can be noticed for motor imagination of feet throughout the whole period 0.4–2 s and for motor imagination of the left hand in the frontal and central areas during preparation/movement initiation period 0.4–0.8 s. Judging by the number of significant electrode locations, there were less changes in the alpha than in the theta band. This is expected because alpha band is a sensory-motor rhythm which should normally be present in people with no pain, i.e. should remain after neurofeedback training.

Fig. 6c shows scalp maps of statistically significant changes in ERS/ERD for the beta band. Difference in ERD can be noticed for all three types of motor imagination across all time windows with no clear spatial locations. In a period *t* = 1.2–2 s there was an increase in ERD (PWP_after-PWP_before) over the parieto-occipital areas for motor imagination of the right hand. Similar to alpha, beta sensory-motor rhythm that is present in people with no pain, and its ERD is therefore not expected to decrease as a result of treatment.

In summary, reduction of ERD is largest over the theta band and smallest over the beta band. The overall reduction (judging by the number of statistically significant electrode locations) is largest for motor imagination of feet.

### 3.2 The effect of neurofeedback training on a difference in ERS/ERD between patients with pain and the other two groups of volunteers

**Fig. 7:**
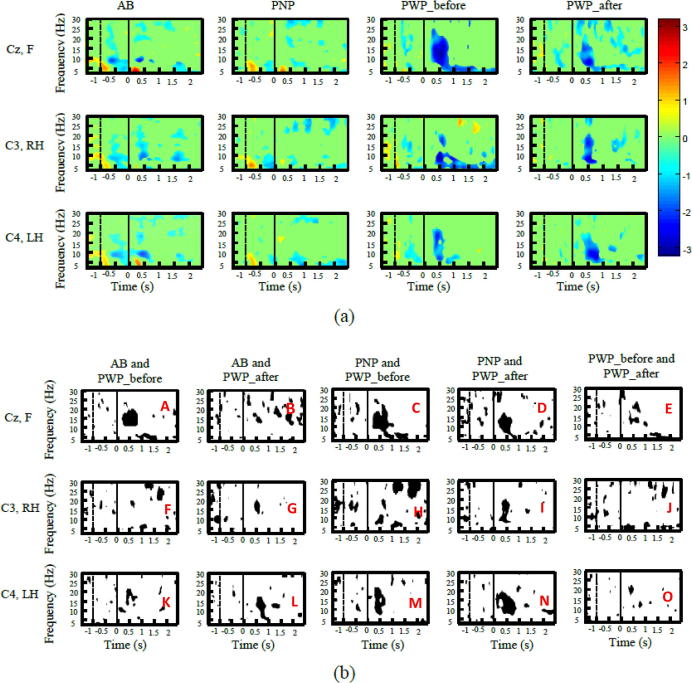
(a) ERD/ERS time frequency maps of four groups (able-bodied (AB): column 1, patients with no pain (PNP): column 2, patients with pain before neurofeedback treatment (PWP_before): column 3, and patients with pain a week after the last neurofeedback session (PWP_after): column 4). Rows present motor imagination of different limbs: the upper row presents Cz location for motor imagination of feet: the middle row presents C3 location for motor imagery of the right hand and he bottom row presents electrode location C4 for motor imagination of the left hand. (b) Comparison of ERSP time frequency maps of four groups (AB and PWP_before: column 1, AB and PWP_after: column 2, PNP and PWP_before: column 3, PNP and PWP_after: column 4, PWP_before and PWP-after: column 5). The order or rows is as in Fig. 6a. Black color presents areas of statistically significant differences (*p* < 0.05 with correction for multiple comparison).

Fig. 7a shows ERD/ERS for AB, PNP and PWP_before and PWP_after for the electrodes located above the primary motor cortex. EEG records signal from both local and distant sources, so recorded signal probably had additional contribution from the premotor cortex and primary sensory cortex, which surround the primary motor cortex. Fig. 7b shows time–frequency maps of statistically significant differences among groups, corrected for multiple comparisons. Larger dark areas correspond to larger, statistically significant differences between groups. Fig. 7b contains multiple subfigures which are all marked with capital letters in red color, for easier referencing.

Fig. 7a shows largest ERD for all groups in the first second post-cue, e.g. during movement planning and initiation. Electrode location Cz was chosen as a representative for motor imagination of feet, electrode C3 for motor imagination of the right hand and C4 for the left hand. While Fig. 3, Fig. 4, Fig. 5 show a sustained ERD for PWP_before and PWP_after on the individual level, in the group analysis this is less visible due to averaging and correction for multiple comparison.

Both PWP_before and PWP_after have stronger ERD (larger dark areas) than the other two groups (AB and PNP), for all three types of imagined movement. However, as shown in Fig. 3, Fig. 4, Fig. 5, ERD is reduced in PWP_after as compared to PWP_before, in particular for motor imagination of feet in the theta band.

Differences between AB and PWP were reduced following NF treatment for motor imagination of feet (Fig. 7b, subfigures A and B). However, situation is not very clear for motor imagination of hands where differences between AB and PWP seem to be larger following NF treatment (subfigures F and G and subfigures K and L). Differences between PNP and PWP were reduced following NF treatment for motor imagination of the feet (subfigures C and D). For motor imagination of the right hand differences were reduced in the higher beta band, in 25–30 Hz (higher than the beta sensory-motor rhythm), but have increased in the alpha/lower beta band in 8–15 Hz (subfigures H and I). For motor imagination of the left hand, differences increased in the alpha/lower beta band (M and N). In summary, the largest changes between PWP and the other two groups, following neurofeedback training can be noticed for motor imagination of feet. Differences decreased in the theta and higher beta band (25–30 Hz) while for motor imagination of hand differences in the alpha band activity increased.

## 4 Discussion

Central neuropathic pain in patients with spinal cord injury is closely related to the increased activity of the sensory-motor cortex during imagined movement (Gustin et al., 2010). Short term reduction of pain by a single session of rTMS restores defective intracortical inhibition (Lefaucheur et al., 2006). Although information is not available, it is most likely that this effect was of a short lasting nature. In this paper we demonstrate that prolonged neurofeedback treatment that leads to longer term reduction of pain also results in decreased activity of sensory-motor cortex, bringing it closer to the activity seen in able bodied people and in spinal cord injured patients with no pain (Stern et al., 2006, Wrigley et al., 2009, Vuckovic et al., 2014). The assessment was performed about a week following the last neurofeedback sessions, so the effect is of a relatively long-lasting nature.

In this study, both assessment and treatment methods have been based on measurement of EEG activity. In principle we could use any other neuromodulatory treatment such as rTMS or tDCS to reduce pain and still use motor imagery as a method to assess the activity of the sensory-motor cortex. Likewise, we could use TMS (as in Lefaucheur et al., 2006) as an assessment tool to evaluate how neurofeedback treatment affects some other aspect of cortical excitability.

The largest overall reduction of activity was noticed for motor imagination of feet and in the theta band, which from previous studies is known as a ‘signature’ of CNP (Sarnthein et al., 2006, Sarnthein and Jeanmonod, 2008, Boord et al., 2008, Jensen et al., 2013b, Vuckovic et al., 2014). Although training was provided from the area of the primary motor cortex, in most cases from electrode location C4 (motor area of the left hand), largest reduction of ERD was noticed in the frontal and parieto-occipital areas. In Vuckovic et al. (2014) we showed that strong wide-spread ERD was a characteristic of patients with CNP. In able-bodied people, largest ERD should be noticed over the central sensory-motor area (Neuper et al., 2006). Reduction of ERD in areas that do not belong to the sensory-motor cortex might therefore indicate normalization of cortical responses during imagined motor task.

When PWP group was compared with AB and PNP groups, over the motor area only, largest changes were noticed for motor imagination of feet, and reflected the overall reduced ERD in PWP_after group. Reduced differences were noticed for ERD of the theta and higher beta band. However for motor imagination of hand, differences in the alpha band ERD between groups increased. This might be related to neurofeedback protocol, training patients to increase the alpha (9–12 Hz) band power. It is of interest that largest differences were noticed for motor imagination of painful limbs, feet, although training was provided from C4. In Hassan et al. (2015) we demonstrated that training from C4 provided wide spread changes of power, and results of the current study (Fig. 6, Fig. 7) indicate that long-lasting cortical changes are not necessarily occurring at the neurofeedback training site. In this study the effect of motor imagination was examined on surface cortical structures only. In our previous study, we showed that the largest changes in the resting state cortical activity occurred in the beta band in deeper cortical structures related to pain processing, including the Dorsolateral Prefrontal Cortex, the Anterior Cingulate Cortex and the Insular Cortex (Hassan et al., 2015). This mechanism of indirect action was also noticed following other neuromodulatory treatments of CNP (rTMS and tDCS) though their longer term effect on pain-related area in SCI patients is not known.

Results of this study indicate a combined effect of changes in the baseline EEG power and reduced activity during motor imagination. It is interesting that although there was some variability in patients’ preferred mental strategy during neurofeedback, some EEG features were commonly related to reduction of pain: reduced beta band baseline power (Hassan et al., 2015) and reduction of theta band ERD over the motor cortex during motor imagination. While there is no clear answer whether changes in EEG are a cause or a consequence of pain, a fact that modulation of resting state EEG through neurofeedback resulted in reduced pain indicate that ‘abnormal’ baseline EEG might precede pain. On the other hand, patients did not practice motor imagination during neurofeedback, so reduced ERD following neurofeedback training might be a consequence of reduced pain.

While there are multiple studies defining resting state EEG signature of CNP, studies looking at the post-effect are rare, and are mainly looking at the short-term effect of a single therapy session. In a recent randomised controlled trial on SCI patients suffering from CNP (Ngernyam et al., 2015), it was demonstrated that a single half an hour tDCS session resulted in significant decrease of pain accompanied with the increase of dominant theta/alpha frequency which stayed significant even 2 days following the treatment. Thus it seems that acute reduction of pain is immediately accompanied by changes of its EEG signatures.

It would be interesting to examine whether other neuromodulatory treatments of CNP that also target primary motor cortex (e.g. rTMS, tDCS) result in a changes in cortical activity in a relaxed state and during motor imagination. This would require longer studies capable of producing longer term changes, as most published studies have less than 10 treatment sessions (Boldt et al., 2014, Nardone et al., 2014, Galhardoni et al., 2015).

A study on another type of neuropathic pain (intercostobrachial pain) (Silva et al., 2014) showed that a single session of both transcutaneous electrical nerve stimulation and acupuncture resulted in the decrease of alpha power that persisted up to 15 min following the treatment. Invasive treatments of CNP are designed to provide much longer lasting treatment effect. It is therefore surprising that although invasive treatments stimulate brain directly, there are not many published results on the long term effect of these treatments on the brain activity, and in particular on the activity of the motor cortex following invasive motor cortex stimulation (Nardone et al., 2014). Although thalamotomy might be no longer a treatment of choice for CNP, Sarnthein et al. (2006) reported the normalization (reduction) of theta activity in 7 patients with mixed origin of CNP a year after thalamotomy that resulted in significant, long lasting, reduction of pain.

Patients from the current study reported reduced pain when we contacted them a month after the last neurofeedback treatment although the intensity of pain slightly increased. During the treatment they learned the mental strategy and were capable of bringing themselves in a similar mental state even without a visual feedback (Hassan et al., 2015). It is therefore hard to say if reduced pain was a consequence of the neurofeedback therapy or it was a results of their independent practice at home. In the future it would be useful having a follow up of EEG measurement over a prolonged period of time, to assess the weather changed in EEG still persist.

Central neuropathic pain is a condition that affects other patient groups such as amputees (Floor, 2002), patients with multiple sclerosis (Osterberg et al., 2005), stroke (Andersen et al., 1995) and patients with Parkinson disease (Beiske et al., 2009). The over-activity of the motor cortex is also noticed in these patient groups (Makin et al., 2013). Improving our understanding of the neural mechanism of non-pharmacological treatments of CNP could also improve the efficacy of treatments.

## Figures and Tables

**Table 1:** Information about patients with pain and about neurofeedback treatment. VNS: visual numerical scale; G: gabapentin; P: pregabalin.

Patient	Level and completeness of injury	Years with injury/pain	Medications	Pain before treatment (VNS)	Pain after treatment (VNS)	Number of neurofeedback training sessions
1	T8 A	7/7	G	6	5	40
2	T7 A	7/7	P	7	5	40
3	T6/T7 D	9/9	P	6	2	40
4	T6/T7 B	25/24	P	9	6	40
5	T8 B	9/9	P	9	6	20

**Table 2:** Information about patients with no pain.

Patient	Level and completeness of injury	Years with injury
1	T7 A	7
2	T7 B	7
3	T12 A	7
4	L1 A	6
5	T2 A	2
6	T5 B	15
7	T11 A	11
8	T4 A	9
9	T7 A	15
10	T7 B	22
